# Does Environmental Innovation Improve Environmental Productivity?—An Empirical Study Based on the Spatial Panel Data Model of Chinese Urban Agglomerations

**DOI:** 10.3390/ijerph17176022

**Published:** 2020-08-19

**Authors:** Junwei Ma, Jianhua Wang, Philip Szmedra

**Affiliations:** 1 Business School, Changshu Institute of Technology, No. 99, 3rd South Ring Road, Changshu 215500, China; mjw@cslg.edu.cn; 2 School of Management, Wuhan University of Science and Technology, No. 947, Peace Avenue, Wuhan 430081, China; 3 Dahlkemper School of Business, Gannon University, Americus, 109 University Squar, Erie, PA 16541, USA; szmedra001@gannon.edu

**Keywords:** environmental innovation, environmental productivity, spatial spillover effect, spatial panel data model, urban agglomeration

## Abstract

Environmental productivity comprehensively measures economic growth and environmental quality. Environmental innovation is considered to be the key to solving economic and environmental problems. Therefore, discussing the impact of environmental innovation on environmental productivity will reveal its economic and environmental effects. This paper measures environmental productivity by value added per unit of pollution emissions (four types of emissions are used) using panel data of 10 Chinese urban agglomerations from 2003 to 2016 to analyze the spatial correlation of environmental productivity, and constructs a spatial panel data model to empirically test the impact of environmental innovation on environmental productivity. It was found that environmental productivity measured by value added per unit of carbon dioxide emissions (gross domestic product (GDP)/CO_2_) had a significant positive spatial spillover effect, and measured by value added per unit of sulfur dioxide emissions (GDP/SO_2_), smoke (dust) emissions (GDP/SDE), and industrial sewage emissions (GDP/IS) had a significant negative spatial spillover effect. Environmental innovation has a significant negative inhibitory effect on environmental productivity measured by GDP/SDE and GDP/IS, but no obvious effect measured by GDP/CO_2_ and GDP/SO_2_. Control variables such as economic development level, industrial agglomeration, foreign direct investment, and endowment structure factor also show significant differences in environmental productivity measured by value added per unit of pollution emissions. In addition, there are significant differences in direct effects of explanatory variables on environmental productivity of local regions and indirect effects on neighboring regions. These differences are also related to the types of pollution emissions. Therefore, policymakers should set different policies for different types of pollution and encourage different types of environmental innovation, so as to achieve reduced pollution emissions and improved environmental productivity.

## 1. Introduction

Achieving sustainable development has prompted researchers and policymakers to focus on the determinants of emissions, such as those of CO_2_, SO_2_, and so on. This allows the development of measures and policies needed to protect the environment and reduce emissions.

Over the past 30 years, due to the limitation of productivity, China’s economic growth has been based on “high investment, high consumption, and high emissions” which has promoted rapid economic development but has also brought serious environmental pollution at the same time [1]. Based on a forecast for 2005–2035, China is to replace the USA as the world’s leading embodied energy consumer in 2027, when its energy consumption per capita will be one quarter of the USA’s [2]. The Global Environmental Performance Index (EPI) ranked China 109th among 180 participating countries and regions in 2016 (it ranked 120th in 2018), which reflects that China’s environmental situation is apparently not optimistic. Because of this, the government has attached great importance to conserving energy and reducing pollution emission, pointing out in “the 13th Five-Year Plan” of China the target of “greatly improving the efficiency of exploiting energy resources, effectively controlling energy consumption, the total amount of carbon emissions, and greatly reducing major pollution emissions.” Achieving this goal is unlikely to be separated from enacting and enforcing environmental regulations and innovations, and the continuous strengthening of those regulations and innovations constitutes an inevitable trend of China’s economic and social development [3,4].

Environmental innovation refers to new products, production processes, services, and management or business methods that will effectively reduce environmental risks, pollution, and other negative impacts in the use of resources throughout the entire life cycle [5], which is considered to be the key to solving economic and environmental problems. Popp et al. [6] pointed out that the decoupling of economic growth and environmental degradation mainly relies on technological improvements to reduce the environmental pressure of production and consumption. Barbieri et al. [7] believed that technological progress to improve environmental quality and reduce environmental pressure will also reduce the cost of achieving environmental goals. The World Intellectual Property Organization (WIPO) forecasts that by 2040, the world’s energy demand will be as much as 30% higher than it is now, and the traditional approach of relying on an expanding energy supply is unsustainable. Innovation in climate-friendly green technology is clearly essential in dealing with energy or environmental issues [8]. Therefore, in theory, environmental innovation may bring about a win-win situation of improved environmental quality and economic growth. WIPO launched an online tool that facilitates the search for patent information related to environmentally sound technology (EST). The tool, combined with WIPO’s International Patent Classification (IPC) system, will help identify existing and emerging green technology. According to this green technology patent search tool, this paper selects the number of green patent applications as the proxy variable for environmental innovation.

Existing studies have mainly analyzed the economic effect of environmental innovation [9], but less attention has been paid to the environmental effect. This is mainly because most researchers usually consider or assume a priori that environmental innovation will certainly improve environmental quality [10]. In fact, environmental innovation may or may not improve environmental quality. For example, Constantini et al. [11] believed that environmental innovation would significantly reduce environmental degradation. Ding et al. [12] also believed that green technology did not play a significant role in environmental protection. Therefore, the real environmental effect of environmental innovation still needs to be investigated in depth. In addition, most of the existing studies analyzed certain aspects of economic or environmental effects of environmental innovation. There is no comprehensive analysis of both, and there is less discussion on the spatial spillover effect. Existing studies have also not distinguished the differences represented by different pollution types.

Environmental productivity is a comprehensive indicator that includes economic growth and environmental quality factors, reflecting the efficiency of environmental utilization. Therefore, analyzing the impact of environmental innovation on environmental productivity can comprehensively evaluate the economic and environmental effects of environmental innovation. Environmental productivity is measured by value added per unit of pollution emissions (four types of pollution emissions are used). This paper reveals different theoretical mechanisms of environmental innovation in economic growth and environmental quality, and constructs a spatial panel data model to empirically test the impact of environmental innovation on environmental productivity, in order to provide theoretical support and empirical evidence for formulating reasonable environmental innovation policies. Compared with existing research, the main innovations of this paper are as follows: First, environmental innovation is accurately measured. Existing studies mostly use the overall national or regional research and development (R&D) investment or patent level to represent environmental innovation, which will exaggerate the impact. According to WIPO’s definition of environmental innovation, this paper uses the classification standard for green patents in the organization’s “International Patent Classification Green List”, which identifies and accounts for the annual number of green patents in urban agglomerations, to be used as the core measurement indicator of environmental innovation. Second, the spatial correlation of environmental productivity among urban agglomerations is analyzed. Third, the spatial panel data model verifies the impact of environmental innovation on environmental productivity, and reveals its economic and environmental effects. Fourth, this paper considers differences in the measurement of environmental productivity by the choice of pollution emissions, and examines differences in the spatial spillover effect of environmental productivity due to different types of emissions. Regarding the choice of pollution emissions, there is greater flexibility. Compared with the previous studies on the single type of pollution emissions, the use of multiple types of pollution emissions and comparative analysis methods applied in this paper is novel. Finally, this paper studies multiple urban agglomerations, which are different from previous studies on single urban agglomeration and cities. Due to the characteristics of high-density spatial agglomeration of urban agglomerations, the spatial panel data models constructed in this paper are novel and suitable, which are conducive to in-depth analysis of the spatial driving mechanisms and spatial effects of high-density agglomeration of urban agglomerations.

The next section of this five-section paper reviews the literature and develops research hypotheses. Section 3 describes empirical methods and materials. Section 4 interprets the results. Section 5 summarizes the major findings and policy implications, then presents limitations and suggestions for future work.

## 2. Literature Review

Environmental innovation includes both economic and environmental effects, which can be expressed in terms of environmental productivity. Environmental productivity reflects the efficiency of environmental utilization, which can simultaneously reveal economic growth and environmental quality.

The concept of environmental productivity was first proposed by Repetto, and it was measured by different methods to reveal the efficiency of environmental utilization [13]. Kortelainen [14] used cutting-edge efficiency techniques and the Malmquist index to construct an environmental productivity index. Bojnec and Papler [15] applied correlation, regression, and multivariate factor analyses to test the associations between the selected structural variables of energy intensity consumption and economic efficiency, and found that the technological intensity of products reduces energy consumption, which was related to the restructuring of energy-intensive industries into more advanced and energy-saving ones with higher value added per unit of product, but with lower energy consumption per unit of product. Farzanegan and Mennel [16] used different pollution indicators to confirm that fiscal decentralization would increase pollution, but better system quality could alleviate this adverse effect, thus confirming that the environment would produce the phenomenon of “competition to the end”. Banzhaf [17] used carbon dioxide emissions as a standard for measuring air quality pollution in the United States and compared and analyzed the degree of environmental pollution control by policies of the US federal and state governments, and found that the federal government’s policies could improve the environmental level, but those of state governments did not show a clear positive effect. Beltran-Esteve and Picazo-Tadeo [18] used data envelopment analysis to estimate the environmental productivity change trends of the transportation industry in 38 countries. Wang and Shen [19] used the general Malmquist–Luenberger (GML) index to measure China’s industrial productivity, including environmental factors, and examined the relationship between environmental regulation and environmental productivity. Zhang and Ye [20] extended the hyperbolic distance function parameter and measured environmental total factor productivity, and decomposed it into environmental efficiency improvements and technological progress. It was found that the environmental efficiency between regions varied greatly, and increased environmental productivity mainly comes from technical progress rather than efficiency improvement. Li et al. [21] applied the metafrontier Malmquist–Luenberger index and a spatial Durbin model to investigate the influence of both local and civil environmental regulations and the spatial spillover effect on green total factor productivity in 273 cities of China. Shen et al. [22] used the threshold model to investigate the nonlinear dynamic influence of different types of environmental regulations on the environmental total factor productivity of industrial sectors. Zhao et al. [23] adopted a super-slacks-based measure model with undesirable outputs to calculate the green economic efficiency in 30 provinces of China, and found that foreign trade dependence and direct investment had significant positive effects. 

Overall, the relevant research mainly focuses on measuring environmental productivity, and few studies discuss the influencing factors. Different from the research focus of scholars, this paper focuses on discussing the mechanism of environmental innovation on environmental productivity, revealing the economic and environmental effects of environmental innovation. We believe that environmental innovation changes environmental productivity by affecting environmental quality and production efficiency. From the perspective of theoretical mechanism, environmental innovation may not only have a positive effect on environmental productivity through environmental quality and production efficiency improvement, but also a negative effect through environmental quality deterioration and profit decline [24].

First, environmental innovation changes environmental productivity by affecting environmental quality. On the one hand, environmental innovation directly affects pollution emissions by effectively reducing them, thereby improving environmental productivity. Song et al. [25] confirmed that improvements in environmental technologies played a dominant role in enhancing China’s environmental total factor productivity. Constantini et al. [11] used data from 1995 to 2009 in 27 European Union (EU) countries to analyze the environmental effect of ecological innovation and found that it inhibited environmental degradation. Ghisetti and Quatraro [10] used green patents to measure environmental innovation, and found that regional departments with higher levels of green technology had better environmental performance. On the other hand, environmental innovation deteriorates environmental quality and reduces environmental productivity through the energy rebound effect. Van Den Berghet et al. [26] found that if environmental innovation improved environmental quality, it could relieve the constraints caused by environmental quality problems to a certain extent. With the use of environmental innovation, environmental quality can be improved, and constraints such as “environmental governance and intensive use of resources and production factors” faced by consumers and enterprises can be reduced. Considering the possible short-sighted behavior of consumers and companies and the high cost of environmental innovation, companies will revert to investing in polluting resources, which will deteriorate the environment. This is a rebound effect. In particular, individuals’ limited rationality makes it difficult to realize that the environmental deterioration caused by changes in their behavior will aggravate the overall rebound effect.

Second, environmental innovation changes environmental productivity by affecting production efficiency. On the one hand, environmental innovation can improve production efficiency and environmental productivity. Porter and Van Der Linde [27] found that environmental innovation improved production efficiency, reduced pollution emissions, and increased competitiveness, which means increased environmental productivity. Ghisetti and Rennings [9] also found that environmental innovation increased corporate competitiveness. On the other hand, environmental innovation requires companies to pay new costs, thereby reducing corporate profits and environmental productivity. For example, Jaffe [28] found that if there was profit in environmental innovation, profit-maximizing companies would inevitably use it to obtain profits, but the actual situation is that companies rarely carry out environmental innovation, which shows that it is more costly, therefore, companies are reluctant to implement it. It may be that the initial cost of environmental innovation is large and uncertain, which will increase the burden on enterprises and reduce environmental productivity compared with general innovation.

In addition, environmental productivity may have a spatial spillover effect. However, traditional non-spatial econometric methods ignore the spatial factors. Although the economic development levels of different regions are quite different, it is undeniable that there are strong interdependence and spatial spillover effects between regions. If the regional economic growth around certain areas is significant, there may also be a significant economic growth trend in those areas, for example, urban agglomerations and economic growth belts. Conventional spatial econometric models, including the spatial autoregressive model (SAR), spatial error model (SEM), and spatial Dubin model (SDM), consider that spatial correlation among variables are more effective and accurate for regression analysis. Costantini et al. [29] pointed out that innovation spillovers would affect environmental performance, the spillover effect was greater than that of innovation, and ignoring space spillovers would lead to biased explanations. Boussemart et al. [30] found that China’s carbon shadow prices are gradually converging, mainly due to the impact of industrial structure changes in eastern China on the central and western regions. Therefore, there may be a significant spatial spillover effect in environmental productivity. If the environmental productivity of a certain area is high, it may affect the environmental productivity of the surrounding area.

Based on the above analysis, this paper proposes the following theoretical hypotheses:

**Hypothesis** **1** **(H1).** *Environmental productivity has a significant spatial spillover effect.*

**Hypothesis** **2** **(H2).** *Environmental innovation affects environmental productivity through environmental quality and production efficiency, but the impact is uncertain.*

**Hypothesis** **3** **(H3).** *The impact of environmental innovation on environmental productivity is related to the type of pollution emissions.*

## 3. Methods and Materials

In this section, we describe the methods, index system, and data sources.

### 3.1. Methods

**Figure 1:**
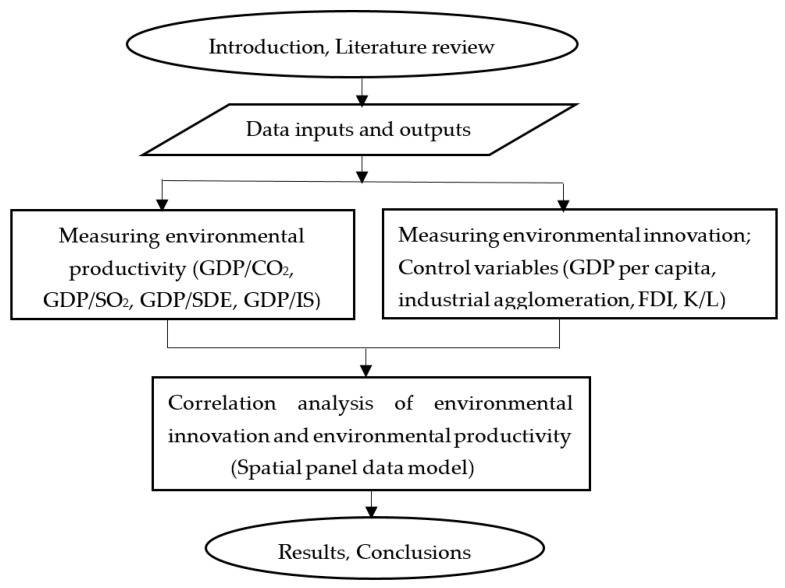
Flow chart of research framework.

According to the mechanism and theoretical hypotheses of environmental innovation in environmental productivity, and considering the differences in environmental productivity measured by value added per unit of pollution emissions, a theoretical and empirical analysis framework is incorporated and checked. In order to achieve this goal, a flow chart of the research framework was constructed, and is shown in Figure 1.

According to the theoretical and empirical analysis framework, this paper constructs the following spatial panel data models:

(1) $${\ln\;\mathrm{EPCD}}_{\mathrm{it}}=\beta w_{i}{\;\ln\;\mathrm{EPCD}}_{t}+\theta {\;\ln\;\mathrm{EI}}_{\mathrm{it}}+{\varphi \;\ln\;X}_{\mathrm{it}}+\mu_{i}+\gamma_{t}+\varepsilon_{\mathrm{it}}$$

(2) $${\ln\;\mathrm{EPSD}}_{\mathrm{it}}=\beta w_{i\;}{\ln\;\mathrm{EPSD}}_{t}+\theta \;{\ln\;\mathrm{EI}}_{\mathrm{it}}+{\varphi \;\ln\;X}_{\mathrm{it}}+\mu_{i}+\gamma_{t}+\varepsilon_{\mathrm{it}}$$

(3) $${\ln\;\mathrm{EPSDE}}_{\mathrm{it}}=\beta w_{i}\;{\ln\;\mathrm{EPSDE}}_{t}+\theta {\;\ln\;\mathrm{EI}}_{\mathrm{it}}+{\varphi \;\ln\;X}_{\mathrm{it}}+\mu_{i}+\gamma_{t}+\varepsilon_{\mathrm{it}}$$

(4) $${\ln\;\mathrm{EPIS}}_{\mathrm{it}}=\beta w_{i}\;{\ln\;\mathrm{EPIS}}_{t}+\theta {\;\ln\;\mathrm{EI}}_{\mathrm{it}}+{\varphi \;\ln\;X}_{\mathrm{it}}+\mu_{i}+\gamma_{t}+\varepsilon_{\mathrm{it}}$$

where ${\ln\;\mathrm{EPCD}}_{\mathrm{it}}$, ${\;\ln\;\mathrm{EPSD}}_{\mathrm{it}}$, ${\;\ln\;\mathrm{EPSDE}}_{\mathrm{it}},{\;\mathrm{and}\;\ln\;\mathrm{EPIS}}_{\mathrm{it}}$ represent environmental productivity of urban agglomeration *i* in the year *t* (as measured by gross domestic product (GDP)/carbon dioxide emissions (CO_2_), GDP/sulfur dioxide emissions (SO_2_), GDP/smoke (dust) emissions (SDE), and GDP/industrial sewage emissions (IS), respectively), $w_{i}{\ln\;\mathrm{EPCD}}_{t},\;\;w_{i}{\ln\;\mathrm{EPSD}}_{t}$,$\;w_{i}{\ln\;\mathrm{EPSDE}}_{t},\;\mathrm{and}\;w_{i}{\ln\;\mathrm{EPIS}}_{t}$ represent the spatial lag and spatial spillover effect of environmental productivity, and $w_{i}$ is the *i*-th row of spatial weight matrix, W. In this paper, the geographic distance matrix of the urban agglomeration is selected to form the spatial weight matrix, W, which is consistent with the general regional spatial correlation law; that is, as the distance between regions gradually expands, the regional correlation gradually weakens. Distance between central cities of the urban agglomeration are calculated using the latitude and longitude of cities announced by the State Bureau of Surveying and Mapping of China. ${\mathrm{EI}}_{\mathrm{it}}$ is environmental innovation of the urban agglomeration *i* in the year *t*. $X_{\mathrm{it}}$ is a matrix of control variables that affect environmental productivity, mainly including factors such as the level of economic development [31,32,33,34,35,36,37], foreign direct investment [38], the degree of industrial agglomeration [39,40,41,42,43], and endowment structure. φ is the corresponding coefficient matrix,$\;\mu_{i\;}\;$ is an individual effect, $\gamma_{t}$ is a time effect, and $\varepsilon_{\mathrm{it}}$ is a random error term. *i* = 1, 2,…, 10, and *t* = 2003, 2004,…, 2016. Calibrated variables in the spatial panel data models are shown in Table 1.

### 3.2. Index System

#### 3.2.1. Environmental Productivity

There are two common methods of measuring environmental productivity. One, according to the definition of environmental productivity, is to use value added per unit of pollution emissions [10], that is, Y/E, where Y is value added and E is pollution emissions. It can be seen that the greater value added per unit of pollution emissions, the higher the environmental efficiency. The other method is to use environmental total factor productivity, and the main measurement methods include the Luenberger productivity index [44], Malmquist–Luenberger productivity index [45], non-radial and non-angle slack-based measure model (SBM) [46,47], and extended Malmquist productivity index [48].

Referring to the research of Ghisetti and Quatraro [10], this paper uses the first method to measure environmental productivity with four pollution emissions: carbon dioxide (CO_2_), sulfur dioxide (SO_2_), smoke (dust) emissions (SDE), and industrial sewage (IS), so environmental productivity is measured by GDP/CO_2_, GDP/SO_2_, GDP/SDE, and GDP/IS. Except for CO_2_, the other pollution emission data of urban agglomerations comes from the China Environmental Statistics Yearbook 2003–2017. The value added uses the real GDP of 2002 prices.

Currently, there are no official uniform data for CO_2_ emissions. This paper calculates the CO_2_ emissions of 10 urban agglomerations in China according to the method provided by the United Nations Intergovernmental Panel on Climate Change (IPCC), as follows:

(5) $$CO_{2}=\sum (EC\times EVF\times CC)\times COF\times 44/12$$

where *CO*_2_ is carbon dioxide emission, *EC* is energy consumption, *EVF* is energy calorific value conversion factor, *CC* is carbon content, and *COF* is carbon oxidation factor. In view of the availability of data, this paper selects eight main energy sources: coal, coke, crude oil, gasoline, kerosene, diesel, fuel oil, and natural gas.

#### 3.2.2. Environmental Innovation

Most studies generally use variables such as R&D expenditures and the number of people engaged in scientific and technological activities to measure environmental innovation. In order to better understand its effect, it is more appropriate to select technological innovation that matches the concept of environmental innovation as its agent variable. This paper selects the number of green patent applications defined by WIPO as the proxy variable for environmental innovation. First, we use the green patent classification defined by WIPO for data cleaning and screening, then, the refined “International Patent Classification (IPC)” number to identify, match, and summarize patents to get the data of green patent applications of 10 urban agglomerations.

#### 3.2.3. Control Index

The level of economic development is expressed in terms of real GDP per capita (PGDP), which measures the impact of regional economic development on environmental productivity. Generally speaking, as the level of economic development increases, environmental productivity will gradually improve. The factor of industrial agglomeration (IA) is expressed by the Krugman specialization index, which measures the impact of industrial agglomeration on environmental productivity. The formula for calculating the Krugman specialization index is

(6) $${\;\mathrm{IA}}_{\mathrm{ij}}=\sum_{k=1}^{n}|X_{\mathrm{ik}}-X_{\mathrm{jk}}|,$$

where ${\;\mathrm{IA}}_{\mathrm{ij}}$ is the Krugman specialization index, i and j are two regions being compared, n is the number of industries, and k = 1, 2, 3…, n and $X_{\mathrm{ik}}$ and $X_{\mathrm{jk}}$ are the proportions of industry k in regions i and j respectively, accounting for the proportion of the entire industry. The value of the index is between 0 and 2. When the industrial structure of two regions is completely the same, the value is 0; when the structure is completely different, the value is 2.

### 3.3. Data Sources

This paper uses spatial panel data from 2003 to 2016 in 10 regions. The “13th Five-Year Plan” issued by the National Development and Reform Commission of China was proposed to promote the sustainable development of some key urban agglomerations. With reference to the Chinese government’s development plan and the consensus of most scholars, this paper selects 10 urban agglomerations as study samples: Beijing–Tianjin–Hebei, Yangtze River Delta, Pearl River Delta, Shandong Peninsula, west coast of the straits, central–southern Liaoning, central plains, middle reaches of Yangtze River, Chengdu–Chongqing, and the central Shaanxi plain (Table 2), which include a total of 122 cities. In 2013, these 10 urban agglomerations had a population of 533 million and total GDP of 38.29 trillion CNY, accounting for 39.2% of the total population and 67.3% of total GDP in China. These urban agglomerations are the most fundamental areas supporting China’s land development and also play a vital role in the country’s participation in global competition. Geographically, they involve three regions in the east, middle, and west of China with gradient differences, and can better represent the economic development level and characteristics of the three regions.

In this paper, most statistical data were derived from the authoritative statistical yearbooks, including the 2004–2017 China Environmental Statistics Yearbook, the 2004–2017 China Urban Statistical Yearbook, and the 2004–2017 China Statistical Yearbook. The data of environmental innovation was obtained according to the green technology patent search tool provided by WIPO. Table 3 reports the sample statistics of environmental productivity, environmental innovation, and other variables in China’s largest urban agglomerations (Table 3).

## 4. Results

In this section, the results are interpreted. We analyze the spatial correlation test of environmental productivity, spatial panel data estimation strategy, spatial estimation and effects of environmental innovation affecting environmental productivity, and the robustness test.

### 4.1. Spatial Correlation Test of Environmental Productivity

Moran’s I is generally used to describe the variables of spatial correlation and reflect the characteristics of the clustering patterns of economic phenomena between regions. This paper also uses Moran’s I to examine the spatial correlation of environmental productivity. The formula is as follows:

(7) $$\mathrm{Moran}’s\;I=\frac{n}{\sum_{i=1}^{n}\sum_{j=1}^{n}W_{ij}}\times \frac{\sum_{i=1}^{n}\sum_{j=1}^{n}W_{ij}\left(X_{i}-\bar{X}\right)\left(X_{j}-\bar{X}\right)}{\sum_{i=1}^{n}{\left(X_{i}-\bar{X}\right)}^{2}}$$

In Equation (7), $X_{i}$ is the observed value of region *i* and $W_{ij}$ is the standardized spatial weight matrix. According to Equation (7), the value of Moran’s I ranges from −1 to 1. At a given significance level, a value greater than 0 indicates a positive correlation, indicating that observations with similar attributes are spatially clustered. On the contrary, it also indicates that observations with different attributes are in a state of aggregation. If the value is close to 0, it indicates that the observations are randomly distributed in space or have no spatial autocorrelation. This paper uses the constructed geographic distance matrix for spatial correlation analysis. Table 4 reports the Moran’s I values of the spatial correlations of environmental productivity in China’s urban agglomerations.

It can be seen that the Moran’s I values of environmental productivity measured by GDP/CO_2_, GDP/SO_2_, GDP/SDE, and GDP/IS are all greater than 0, and all pass the significance test. This shows that no matter which type of pollution emission is used, the environmental productivity of Chinese urban agglomerations has a significant positive spatial correlation, so hypothesis H1 passes the test.

### 4.2. Spatial Panel Data Estimation Strategy

In order to accurately study the impact of environmental innovation on environmental productivity and its spatial effect, it is necessary to further conduct spatial metrological inspection. Choosing a suitable spatial panel data estimation method is helpful to accurately reflect the causes of spatial dependence and the effects of different spatial association mechanisms. This paper refers to Elhorst’s testing ideas and uses a combination of “specific-to-general” and “general-to-specific” methods to test the spatial panel data model [49]. First, according to the specific-to-general test ideas, we estimate the nonspatial model and use the Lagrange multiplier (LM) method to test whether to use the spatial autoregressive model (SAR) or spatial error model (SEM). If maximum likelihood estimation spatial lag (LM-lag) passes the test instead of maximum likelihood estimation spatial error (LM-err), the SAR model is selected, and vice versa; if both LM-lag and LM-err pass the test, further comparison between Robust-LM-lag and Robust-LM-err is required. If Robust-LM-lag passes the test instead of Robust-LM-err, the SAR model is selected, and vice versa. Second, if the non-spatial effect model is rejected and there is a fixed effect in space or time, then the spatial Dubin model (SDM) needs to be estimated according to the “general-to-specific” test idea, and the likelihood ratio test (LR-test) is used to measure whether the model has a spatial fixed effect (SFE) or time fixed effect (TFE). Third, Hausman’s test is performed to further determine whether the SDM uses a fixed effect or random effect estimation method. Finally, the Wald or LR test is used to determine whether the SDM will be simplified to SAR or SEM. If both of the above hypotheses are rejected, the SDM is the best choice for estimating the spatial panel data model. If the first hypothesis cannot be rejected and the LM (R-LM) also points to the SAR model, then the SAR is a better spatial panel data model. If the second hypothesis cannot be rejected and LM (R-LM) also points to the SEM model, then SEM is the optimal model in spatial panel data estimation. If the model tests by LM (R-LM) and Wald (or LR) are inconsistent, then the SDM is more suitable for estimating the spatial panel data model, because it is a generalized form of both SAR and SEM. Table 5, Table 6, Table 7 and Table 8 report the test results of the spatial panel data model of environmental productivity measured by GDP/CO_2_, GDP/SO_2_, GDP/SDE, and GDP/IS.

According to the selection criteria [50,51] and estimation results of the spatial panel data model, the spatial panel data model of environmental productivity measured by GDP/CO_2_ is suitable for selecting the SDM model of random effect, and the model measured by GDP/SO_2_, GDP/SDE, and GDP/IS is suitable for selecting the SDM model of the space-time double fixed effect.

### 4.3. Estimation of Spatial Panel Data Model of Environmental Ennovation Affecting Environmental Productivity

The spatial panel data model estimation results of environmental innovation on environmental productivity (as measured by GDP/CO_2_, GDP/SO_2_, GDP/SDE, and GDP/IS) are as shown in Table 9.

From the spatial autoregressive coefficients (ρ) and significance test results in each equation, the spatial autoregressive coefficients of environmental productivity measured by GDP/CO_2_, GDP/SO_2_, GDP/SDE, and GDP/IS are all significant at 1%, indicating that there is an obvious spatial dependence relationship between environmental productivity of urban agglomerations, so hypothesis H1 passes the test. Among them, the productivity measured by GDP/CO_2_ has a significantly positive spatial spillover effect, indicating that the higher environmental productivity in this region is conducive to improved productivity in neighboring regions. This may be due to the higher environmental productivity of the local region showing a strong diffusion effect, which is conducive to improved productivity in neighboring regions. The environmental productivity measured by GDP/SO_2_, GDP/SDE, and GDP/IS has a significantly negative spatial spillover effect, indicating that higher productivity in the local region is not conducive to improved productivity in neighboring regions. This may be due to the “siphon” effect of higher environmental productivity in the local region, which causes a large amount of resources to flow into areas with high productivity, which is not conducive to improved productivity in neighboring areas.

From the perspective of the impact of environmental innovation on environmental productivity, there are significant differences, as measured by GDP/CO_2_, GDP/SO_2_, GDP/SDE, and GDP/IS. Environmental innovation has a positive effect on environmental productivity, as measured by GDP/CO_2_, but it does not pass the significance test, indicating no significant impact on Chinese urban agglomerations. Environmental innovation has a negative inhibitory effect on environmental productivity, as measured by GDP/SO_2_, but it fails the significance test, indicating no significant impact on Chinese urban agglomerations. Environmental innovation has a significant negative inhibitory effect on environmental productivity, as measured by GDP/SDE, indicating that its negative effect on smoke (dust) emissions exceeds the positive effect on economic growth. Environmental innovation has a significant negative inhibitory effect on environmental productivity, as measured by GDP/IS, indicating that its negative effect on industrial sewage emissions exceeds the positive effect on economic growth. It can be seen that the impact of environmental innovation on environmental productivity is inconsistent. Environmental innovation has a significant negative inhibitory effect on environmental productivity, as measured by GDP/SDE and GDP/IS, and has no obvious effect, as measured by GDP/CO_2_ and GDP/SO_2_. Hypotheses H2 and H3 pass the test. This is mainly due to the selection of different types of pollution emissions and the measurement of different types of environmental productivity. This result is consistent with those of Van Den Berghet et al. [26] and Jaffe [28]. This is mainly because environmental innovation deteriorates environmental quality through the energy rebound effect, or the high cost makes enterprises unwilling to adopt environmental innovation, which is not conducive to improved productivity.

In addition, there are significant differences in the impact of control variables such as economic development, industrial agglomeration, foreign direct investment (FDI), and structural factors on environmental productivity measured by GDP/CO_2_, GDP/SO_2_, GDP/SDE, and GDP/IS. The level of economic development (PGDP) has a significant positive effect on environmental productivity measured by GDP/CO_2_, GDP/SO_2_, and GDP/IS, indicating that its positive effect on economic growth exceeds the negative effect on CO_2_, SO_2_, and IS emissions. PGDP has a positive effect on environmental productivity measured by GDP/SDE, but it fails the significance test. Industrial agglomeration (IA) has a significant positive effect on environmental productivity measured by GDP/IS, indicating that its positive effect on economic growth exceeds the negative impact on IS emissions. IA has a positive effect on environmental productivity measured by GDP/CO_2_, GDP/SO_2_, and GDP/SDE, but it does not pass the significance test. This may be due to the recycling of resources and proliferation of clean technology within industrial clusters reducing pollution emissions. Foreign direct investment (FDI) has a significant positive effect on environmental productivity measured by GDP/SO_2_ and GDP/SDE and does not support the “pollution paradise” hypothesis. The impact of FDI on environmental productivity measured by GDP/CO_2_ and GDP/IS is not significant, so it does not support the “pollution paradise” hypothesis. This may be because the host country’s economic development level, political stability, and legal integrity are the key factors determining its FDI level, and environmental regulatory policies have almost no effect. The capital-labor ratio (K/L), reflecting the endowment structure, has a significant positive effect on environmental productivity measured by GDP/SDE, indicating that the positive effect of the technological progress and environmental innovation of capital-intensive enterprises on economic growth exceeds the negative effect on SDE. K/L has a significant negative effect on environmental productivity measured by GDP/IS, indicating that the negative impact of technological progress and environmental innovation of capital-intensive enterprises on IS emissions exceeds its positive effect on economic growth. K/L has a negative effect on the environmental productivity measured by GDP/CO_2_ and GDP/SO_2_, but it fails the significance test. The possible reason for this is that the economic structure of the urban agglomeration is transforming from labor-intensive industries, which tend to be lightly polluting, to capital-intensive industries, which tend to be heavily polluting.

**Figure 2:**
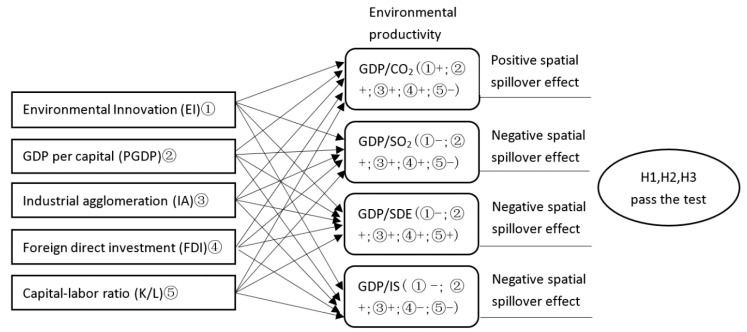
Research results (+, positive; −, negative).

The results of environmental innovation on environmental productivity can be seen in Figure 2.

### 4.4. Direct and Spillover Effects of Environmental Innovation and Other Variables on Environmental Productivity

In order to determine whether environmental innovation and other variables have a significant spatial spillover effect on environmental productivity, this paper further estimates their direct and indirect effects in various spatial panel data models based on the parameter estimation results in Table 9. Among them, the direct effect reflects the impact of explanatory variables such as environmental innovation in the local region on environmental productivity, and the indirect effect indicates the spatial impact of environmental innovation in the local region on the environmental productivity of neighboring regions, which reflects the spatial spillover effect. The estimated results of direct and indirect effects are shown in Table 10.

It can be seen that the direct and indirect effects of explanatory variables on environmental productivity are significantly different, and the direction of influence is related to the type of pollution emissions.

For environmental productivity measured by GDP/CO_2_, the direct effect of environmental innovation (ln EI) does not pass the significance test, and the indirect effect is significantly positive, indicating that environmental innovation has no significant impact on environmental productivity measured by GDP/CO_2_ in the local region, but a positive spatial spillover effect on neighboring regions. The direct effect of the level of economic development (ln PGDP) is significantly positive, and the indirect effect is significantly negative, indicating that it has a significant positive effect on environmental productivity measured by GDP/CO_2_ in the local region, but a negative spatial spillover effect for neighboring regions. Neither the direct nor indirect effect of industrial agglomeration (ln IA) passes the significance test, indicating that it has no significant impact on environmental productivity measured by GDP/CO_2_ in the local region and neighboring regions. The direct effect of foreign direct investment (ln FDI) does not pass the significance test, and the indirect effect is significantly negative, indicating that it has no significant impact on environmental productivity measured by GDP/CO_2_ in the local region, but a negative spatial spillover effect in neighboring regions. The direct and indirect effects of structural factors (ln (K/L)) do not pass the significance test, indicating that the capital-labor ratio has no significant impact on environmental productivity measured by GDP/CO_2_ in the local region and neighboring regions.

Similarly, according to the parameter estimation results of each explanatory variable, it can be analyzed whether each explanatory variable has a significant impact on environmental productivity measured by GDP/SO_2_, GDP/SDE, and GDP/IS in the local region and neighboring areas.

### 4.5. Robustness Test

The result of the spatial panel data model is affected by the setting of the spatial weight matrix. In this paper, the geographic distance matrix is selected as the spatial weight matrix. In order to test the robustness of the above spatial metrology estimation results, we use the economic distance matrix and the economic and geographic distance nested matrices as the new spatial weight matrix. The new spatial weight matrix construction method is as follows.

First is the economic distance matrix, which can be set as:

(8) $$W_{e}=1/\left|\bar{Q_{i}}-\bar{Q_{j}}\right|$$

where $W_{e}$ is the economic distance matrix, and $\bar{Q_{i}}$ and $\bar{Q_{j}}$ represent the GDPs per capita of regions *i* and *j* in 2003–2016.

Second is the geographic and economic distance nested matrix. This is obtained by selecting different weights for weighting. At the same time, considering the geographic proximity of the spatial units and their economic relationship, it can more fully characterize the association between spatial units. The geographic and economic distance nested matrix ($W_{d-e}$) is set as follows:

(9) $$W_{d-e}=\varphi W_{d}+\left(1-\varphi \right)W_{e}$$

where $W_{d}$ is the geographic distance matrix, $W_{e}$ is the economic distance matrix, and *φ* ∈ (0,1), which represents the proportion of the geographic matrix.

Considering new spatial weight matrices such as the economic distance matrix and economic and geographic distance nested matrix, this paper separately estimates the spatial Dubin model (SDM). The parameter estimates of the explanatory variables under the three spatial weight matrices are basically consistent, which verifies the robustness of the estimation results.

## 5. Conclusions

Based on panel data from 2003 to 2016, this paper disscussed the mechanism of environmental innovation on the environmental productivity of 10 urban agglomerations in China based on the spatial Dubin model (SDM) of the space-time double fixed effect.

From the results of SDM estimation, environmental productivity has a significant spatial spillover effect, but the direction of the impact is related to pollution emissions. Among them, environmental productivity measured by GDP/CO_2_ has a significant positive spatial spillover effect, and that measured by GDP/SO_2_, GDP/SDE, and GDP/IS has a significant negative spatial spillover effect.

From the perspective of the impact of environmental innovation on environmental productivity, there are significant differences. Among them, environmental innovation has a significant negative inhibitory effect on environmental productivity measured by GDP/SDE and GDP/IS, and no obvious effect on productivity measured by GDP/CO_2_ and GDP/SO_2_. This shows that environmental innovation does not effectively reduce smoke (dust) and industrial sewage emissions while value added remains unchanged. Environmental innovation is conducive to reducing carbon dioxide emissions while value added remains unchanged, but it fails the significance test. This also shows that environmental innovation is not effective in reducing all types of pollution emissions, and the design of environmental innovation policies should distinguish the differences in pollution emissions. Control variables such as economic development level, industrial agglomeration, foreign direct investment, and endowment structure factors also have significant differences in environmental productivity. In addition, the direct effects of explanatory variables on environmental productivity of the local region and the indirect effects on productivity of neighboring regions also have significant differences. These differences are related to the type of pollution emissions.

In view of the above conclusions, this paper proposes policy recommendations: First, give full play to the economic and environmental effects of environmental innovation and increase environmental productivity so as to achieve sustainable economic and environmental development. Second, environmental innovation cannot only be carried out technologically, but also requires social, economic, and business model innovation, as well as the cultivation of global citizenship, i.e., a clearer understanding of the impact of various environmental policies. Third, the types of pollution emissions cannot be ignored. It is necessary to set different policies for different pollution emissions, and encourage different types of environmental innovation in order to achieve targeted emission reduction. Fourth, the impact of economic development level, industrial agglomeration, foreign direct investment, and endowment structure on environmental productivity should be considered. Finally, it is necessary to consider regional factors and not adopt a one-size-fits-all environmental policy. It should be based on regional realities and reducing different types of pollution emissions in a targeted manner to improve environmental productivity.

However, this paper is somewhat limited and further research is needed. Different methods should be used to measure differences in environmental productivity. In this paper, value added per unit of pollution emissions is used to represent environmental productivity. Subsequent studies may consider total factor productivity measured by different methods to represent environmental productivity, distinguishing between static and dynamic productivity. Also, indicators that affect environmental productivity should be selected. Subsequent research can select different indicators, find out the key control variables, and avoid the subjectivity of indicator selection so as to improve the accuracy of evaluation and the persuasiveness of the research conclusions.

## Figures and Tables

**Table 1:** Calibrated variables. GDP, gross domestic product.

Variables		Abbreviation	Description
Explained variables	Environmental productivity	EPCD	GDP per unit of carbon dioxide emissions (GDP/CO_2_)
		EPSD	GDP per unit of sulfur dioxide emissions (GDP/SO_2_)
		EPSDE	GDP per unit of smoke (dust) emissions (GDP/SDE)
		EPIS	GDP per unit of industrial sewage emissions (GDP/IS)
Explanatory variables	Environmental innovation	EI	Number of green patent applications
	GDP per capita	PGDP	GDP per capita
	Industrial agglomeration	IA	Industrial agglomeration index
	Foreign direct investment	FDI	Amount of foreign direct investment
	Capital-labor ratio	K/L	Capital-labor ratio, indicating the endowment structure

**Table 2:** Definition of China’s largest urban agglomerations.

Regions	Cities
Yangtze River Delta	Shanghai, Nanjing, Hangzhou, Suzhou, Wuxi, Changzhou, Zhenjiang, Yangzhou, Taizhou, Nantong, Jiaxing, Huzhou, Ningbo, Shaoxing, Zhoushan, Taizhou
Pearl River Delta	Guangzhou, Shenzhen, Zhuhai, Foshan, Huizhou, Zhaoqing, Jiangmen, Dongguan, Zhongshan
Beijing–Tianjin–Hebei	Beijing, Tianjin, Tangshan, Langfang, Baoding, Qinhuangdao, Shijiazhuang, Zhangjiakou, Chengde, Zhangzhou
Central–southern Liaoning	Shenyang, Dalian, Anshan, Fushun, Benxi, Fuxin, Panjin, Dandong, Liaoyang, Tieling, Huludao, Yingkou, Jinzhou
Shandong Peninsula	Jinan, Qingdao, Yantai, Weihai, Rizhao, Dongying, Weifang, Zibo
West Coast of the Straits	Fuzhou, Xiamen, Zhangzhou, Quanzhou, Putian, Ningde
Central Plains	Zhengzhou, Luoyang, Kaifeng, Xinxiang, Jiaozuo, Xuchang, Jiyuan, Pingdingshan, Weihe
Middle Reaches of Yangtze River	Wuhan, Changsha, Nanchang, Huangshi, Ezhou, Xiaogan, Huanggang, Xianning, Xiangyang, Yichang, Jingzhou, Jingmen, Zhuzhou, Xiangtan, Yueyang, Yiyang, Changde, Hengyang, Loudi, Jiujiang, Jingdezhen, Yingtan, Xinyu, Yichun, Pingxiang, Shangrao, Fuzhou
Central Shaanxi Plain	Xi’an, Xianyang, Tongchuan, Baoji, Weinan
Chengdu–Chongqing	Chengdu, Chongqing, Deyang, Mianyang, Yibin, Leshan, Zhangzhou, Nanchong, Zigong, Meishan, Neijiang, Suining, Guang’an, Ya’an, Ziyang, Dazhou

**Table 3:** Sample statistical values of variables of China’s largest urban agglomerations (as mean, standard deviation (SD), minimum (Min), and maximum (Max)).

Variables	Mean	SD	Min	Max
EPCD (GDP/CO_2_, RMB 10,000/ton)	0.4521	0.2286	0.1760	1.2480
EPSD (GDP/SO_2_, RMB 10,000/ton)	0.0437	0.0465	0.0035	0.2846
EPSDE (GDP/SDE, RMB 10,000/ton)	0.0997	0.0873	0.0088	0.5511
EPIS (GDP/IS, RMB 10,000/ton)	0.1717	0.1287	0.0233	0.6899
EI (green patents, number)	5438	8322	137	53,168
PGDP (GDP per capita, RMB yuan/person)	52,076	39,094	7292	203,485
IA (industrial agglomeration index, 0–2)	0.3640	0.0736	0.2008	0.5815
FDI (foreign direct investment, USD 100 million)	357	638	5	2418
K/L (capital-labor ratio, RMB 10,000/labor)	11.1146	8.1496	1.4002	39.7765

**Table 4:** Spatial autocorrelation Moran’s I of environmental productivity of Chinese urban agglomerations.

Environmental Productivity	GDP/CO_2_	GDP/SO_2_	GDP/SDE	GDP/IS
Moran’s I	0.393	0.617	0.221	0.546
*p*-value	0.000	0.000	0.000	0.000

**Table 5:** Spatial panel data model test (GDP/CO_2_) under the geospatial matrix. SAR, spatial autoregressive model; SEM, spatial error model; SDM, spatial Dubin model; LM, Lagrange multiplier; SFE, spatial fixed effect; TFE, time fixed effect; LR, likelihood ratio.

	Method	Null Hypothesis	Statistic	*p*-Value	Result
SAR and SEM tests	LM-lag	No spatial lag	84.954	0.000	Reject
	R-LM-lag	No spatial lag	78.267	0.000	Reject
	LM-err	No spatial error effect	8.418	0.004	Reject
	R-LM-err	No spatial error effect	1.731	0.188	Accept
SDM fixed effect test	SFE-LR	No spatial fixed effect	68.060	0.000	Reject
	TFE-LR	No fixed time effect	331.460	0.000	Reject
	STFE-LR	No double fixed effect	199.308	0.000	Reject
Hausman test of SDM	Hausman	Random effect model	0.980	0.964	Accept
Simplified test of SDM	Wald-lag	SDM can be weakened to SAR	39.790	0.000	Reject
	LR-lag	SDM can be weakened to SAR	33.410	0.000	Reject
	Wald-err	SDM can be weakened to SEM	27.690	0.000	Reject
	LR-err	SDM can be weakened to SEM	27.810	0.000	Reject

**Table 6:** Spatial panel data model test (GDP/SO_2_) under the geospatial matrix.

	Method	Null Hypothesis	Statistic	*p*-Value	Result
SAR and SEM tests	LM-lag	No spatial lag	9.479	0.002	Reject
	R-LM-lag	No spatial lag	5.804	0.016	Reject
	LM-err	No spatial error effect	14.853	0.000	Reject
	R-LM-err	No spatial error effect	11.178	0.001	Reject
SDM fixed effect test	SFE-LR	No spatial fixed effect	78.850	0.000	Reject
	TFE-LR	No fixed time effect	97.060	0.000	Reject
	STFE-LR	No double fixed effect	8.090	0.000	Reject
Hausman test of SDM	Hausman	Random effect model	−6.850	0.000	Reject
Simplified test of SDM	Wald-lag	SDM can be weakened to SAR	29.900	0.000	Reject
	LR-lag	SDM can be weakened to SAR	23.760	0.002	Reject
	Wald-err	SDM can be weakened to SEM	22.110	0.001	Reject
	LR-err	SDM can be weakened to SEM	18.380	0.003	Reject

**Table 7:** Spatial panel data model test (GDP/SDE) under the geospatial matrix.

	Method	Null Hypothesis	Statistic	*p*-Value	Result
SAR and SEM tests	LM-lag	No spatial lag	5.001	0.025	Reject
	R-LM-lag	No spatial lag	1.103	0.294	Accept
	LM-err	No spatial error effect	15.636	0.000	Reject
	R-LM-err	No spatial error effect	11.739	0.001	Reject
SDM fixed effect test	SFE-LR	No spatial fixed effect	84.300	0.000	Reject
	TFE-LR	No fixed time effect	154.320	0.000	Reject
	STFE-LR	No double fixed effect	5.195	0.000	Reject
Hausman test of SDM	Hausman	Random effect model	−0.680	0.000	Reject
Simplified test of SDM	Wald-lag	SDM can be weakened to SAR	80.620	0.000	Reject
	LR-lag	SDM can be weakened to SAR	60.320	0.000	Reject
	Wald-err	SDM can be weakened to SEM	65.720	0.000	Reject
	LR-err	SDM can be weakened to SEM	53.990	0.000	Reject

**Table 8:** Spatial panel data model test (GDP/IS) under the geospatial matrix.

	Method	Null Hypothesis	Statistic	*p*-Value	Result
SAR and SEM tests	LM-lag	No spatial lag	5.705	0.017	Reject
	R-LM-lag	No spatial lag	5.964	0.015	Reject
	LM-err	No spatial error effect	0.006	0.939	Accept
	R-LM-err	No spatial error effect	0.264	0.607	Accept
SDM fixed effect test	SFE-LR	No spatial fixed effect	110.780	0.000	Reject
	TFE-LR	No fixed time effect	213.750	0.000	Reject
	STFE-LR	No double fixed effect	137.080	0.000	Reject
Hausman test of SDM	Hausman	Random effect model	15.960	0.007	Reject
Simplified test of SDM	Wald-lag	SDM can be weakened to SAR	139.790	0.000	Reject
	LR-lag	SDM can be weakened to SAR	86.420	0.000	Reject
	Wald-err	SDM can be weakened to SEM	88.240	0.000	Reject
	LR-err	SDM can be weakened to SEM	68.910	0.000	Reject

**Table 9:** Panel data model estimation results.

	(1)	(2)	(3)	(4)
Environmental Productivity	GDP/CO_2_	GDP/SO_2_	GDP/SDE	GDP/IS
Model	SDM Random Effect	SDM Space-Time Effect	SDM Space-Time Effect	SDM Space-Time Effect
ln EI	0.0132	−0.0082	−0.2815 *	−0.1558 **
	(0.33)	(−0.09)	(−1.70)	(−2.52)
ln PGDP	0.5995 ***	1.2847 ***	0.2975	2.2249 ***
	(5.15)	(5.37)	(0.68)	(13.90)
ln IA	0.0885	0.1067	0.2102	0.2105 **
	(1.30)	(0.77)	(0.84)	(2.22)
ln FDI	0.0239	0.0921 **	0.2654 ***	−0.0208
	(1.18)	(2.17)	(3.49)	(−0.73)
ln (K/L)	−0.0570	−0.1510	0.6836 *	−0.7092 ***
	(−0.54)	(−0.71)	(1.80)	(−4.88)
ρ	0.3394 ***	−0.9223 ***	−0.6636 ***	−0.6622 ***
	(3.04)	(−5.29)	(−3.48)	(−4.04)
log-lik	133.4613	82.1149	5.1950	137.0797
R2	0.9361	0.8354	0.5864	0.7228

Note: ***, **, and * indicate significance at the 1%, 5%, and 10% level, and log-lik is log-likelihood.

**Table 10:** Direct and spillover effects of variables on environmental productivity.

Effect Type	Environmental Productivity	GDP/CO_2_	GDP/SO_2_	GDP/SDE	GDP/IS
	Model Variables	SDM Random Effect	SDM Space-Time Effect	SDM Space-Time Effect	SDM Space-Time Effect
Direct effect	ln EI	0.0285	0.0704	−0.2398	−0.1363 **
		(0.71)	(0.76)	(−1.50)	(−2.30)
	ln PGDP	0.5649 ***	1.2172 ***	−0.2231	1.8626 ***
		(4.84)	(5.19)	(−0.56)	(11.89)
	ln IA	0.0813	0.2811 **	0.3515	0.2154 **
		(1.13)	(2.19)	(1.58)	(2.55)
	ln FDI	0.0104	0.0331	0.1575 **	−0.0200
		(0.48)	(0.71)	(1.97)	(−0.71)
	ln (K/L)	−0.0387	−0.1656	0.5734	−0.4185 ***
		(−0.38)	(−0.68)	(1.41)	(−2.83)
Indirect effect	ln EI	0.2726 **	−0.3605	−0.2548	−0.1390
		(2.35)	(−1.59)	(−0.57)	(−0.81)
	ln PGDP	−0.6124 **	0.2397	3.5730 ***	2.6328 ***
		(−2.15)	(0.49)	(3.84)	(6.05)
	ln IA	−0.2715	−0.7988 **	−0.8536	0.0356
		(−0.99)	(−2.24)	(−1.26)	(0.14)
	ln FDI	−0.2485 **	0.2863 ***	0.7776 ***	−0.0123
		(−2.41)	(2.82)	(3.87)	(−0.17)
	ln (K/L)	0.2261	0.1391	1.0268 **	−2.0992 ***
		(0.77)	(0.30)	(1.15)	(−5.02)

Note: ***, and ** indicate significance at the 1%, and 5% level.
